# Review of "The Ten Most Wanted Solutions in Protein Bioinformatics", by Anna Tramontano

**DOI:** 10.1186/1475-925X-5-1

**Published:** 2006-01-03

**Authors:** Kay C Wiese

**Affiliations:** 1 School of Computing Science, Simon Fraser University, 15^th ^Floor, Central City Tower, 13450 102^nd ^Ave, Surrey, B.C., V3T 5X3, Canada

Bioinformatics can be defined as the application of computational tools to manage and analyze biological data, typically at the molecular level. The field of Computational Biology aims at the mathematical or computational modeling of biological processes, such as protein folding or protein-protein interaction. Many books that include the title bioinformatics or computational biology (BCB) actually present a mixture of the two fields. It is sometimes not possible to separate the two as for a single problem such as protein folding both bioinformatics techniques such as similarity search of existing protein databases as well as computational biology is used in the form of mathematically modeling the protein structure via thermodynamic models that are optimized via a computational technique.

Books in BCB can be categorized broadly into application tools books, which describe existing applications (typically available on the WWW) to solve a biological problem [1]. There are also books that do not offer any computational solutions, but should be considered valuable, as they describe a biological domain [2]. The remaining category offers detailed mathematical, statistical, and computational approaches for problems in BCB [3,4] and books that focus on specific computational approaches [5].

The book reviewed here is different in the sense that it focuses on a single domain, namely protein bioinformatics. Ten problems in this domain are identified and discussed in 10 Chapters. The book starts with an introduction to proteins and their structure which is helpful for the uninitiated.

Chapter 1 discusses the basic problem of *Protein Sequence Alignment *and provides a biological motivation based on the evolution of proteins. The standard algorithms for global (Needleman Wunsch) and local alignment (Smith Waterman) are presented. Chapter 2 discusses *Feature Prediction *of proteins including features for secondary structure, active sites, binding ligands or sites being recognized by enzymes. In this context hidden markov models, neural networks and support vector machines are discussed. The important principles of specificity and sensitivity are also introduced. In Chapter 3 the challenges of Protein Function prediction are discussed including the problem of absence of standards in new protein naming and protein function annotation. Another point of caution raised is the issue of error propagation in biological networks due to transferring functional annotations by similarity. *Protein Structure Prediction *is discussed in Chapter 4, which covers energy based modeling of protein structures and the limitations of such approaches, mainly based on the imperfections due to the classical approximation of quantum-mechanical interactions between protein atoms. In addition, a number of computational approaches to search the protein conformational space are discussed. Chapter 5 reviews *Membrane Proteins *and is more biological and less technically oriented than previous chapters. The topic of Chapter 6 is *Functional Site Identification *and the detection of functional sites in proteins and structural superposition are presented. Chapter 7 discusses *Protein-Protein Interaction *and explores sequence-based, structure based and experimental methods for detecting protein-protein interactions. The scoring of docking solutions and the CAPRI experiment are discussed as well. A related problem is presented in Chapter 8, which focuses on *Protein-Small Molecule Interaction*. A number of methods are presented and the implications to the virtual screening of drug compounds for the drug discovery process are discussed. Chapter 9 elaborates on *Protein Design *to synthesize proteins that do not necessarily exist in nature. This is also known as the inverse protein folding problem. Intuitive design, lattice models and automatic models are touched upon. The book concludes with a discussion of *Protein Engineering *in Chapter 10, which deals with engineering novel properties into naturally occurring proteins.

Each chapter offers an introduction to the problem and discusses a number of solutions or solution attempts. It is nice that for proposed solutions either the biological rationale is given, or it is explained how the model's deviation from the biological reality affects the proposed solution. A summary of the reliability of the present methods, suggestions for promising avenues and a list of suggested readings conclude each chapter. The author needs to be commended for fairly discussing a great variety of computational approaches including dynamic programming, hidden markov models, monte carlo methods, simulated annealing, genetic algorithms, neural networks, and heuristic based methods including "intuitive design" as well as for discussing their benefits and drawbacks on selected problems. One of the clear strengths of the book is that it cautions us to accurately test the reliability of methods, assess the appropriateness of approximations and to be aware of the limitations of data produced in wet-lab experiments.

This book is geared towards graduate students or bioinformatics researchers at an intermediate level. It is useful to people from either a life science background or a computational background and a bit of exposure to both areas is helpful. The book is comprehensive and succinct; people on busy schedules can read it in a short period of time. Of course, due to being succinct, it does not provide all the technical details of all discussed computational methods, but I do not see this as a drawback, because it does a great job at drawing interconnections between the related problems and related approaches. One reservation to be noted is that not all illustrations are of the same high quality. Particularly the high quality illustrations from the color insert are reproduced in grayscale where they are discussed in the text and this renders many of them hard to interpret. I believe that it would have been better to simply refer the reader to the color insert. Another issue is that the number of human genes is given as 50,000 (p. 53) and this is dated as more recent estimates are closer to below 30,000 [6]. Nevertheless, these are minor issues, and I believe that it is a great book. I will definitely include it as a recommended text for one of my Bioinformatics Graduate courses. I highly recommend this text to anyone interested in protein bioinformatics.
